# Functional Outcomes and Safety Profile of Trans-Foveal Subthreshold Micropulse Laser in Persistent Central Serous Chorioretinopathy

**DOI:** 10.3390/life13051194

**Published:** 2023-05-16

**Authors:** Peter Kiraly, Maja Šuštar Habjan, Jaka Smrekar, Polona Jaki Mekjavić

**Affiliations:** 1 Eye Hospital, University Medical Centre Ljubljana, 1000 Ljubljana, Slovenia; peter.kiraly20@gmail.com (P.K.);; 2 Oxford Eye Hospital, Oxford University Hospitals NHS Foundation Trust, Oxford OX3 9DU, UK; 3 Faculty of Medicine, University of Ljubljana, 1000 Ljubljana, Slovenia; 4 Faculty of Mathematics and Physics, University of Ljubljana, 1000 Ljubljana, Slovenia; 5 Jožef Stefan Institute, 1000 Ljubljana, Slovenia

**Keywords:** central serous chorioretinopathy, CSC, subthreshold micropulse laser, safety profile, contrast sensitivity, microperimetry, mfERG

## Abstract

Our study evaluated visual function changes after subthreshold micropulse laser (SML) treatment in persistent central serous chorioretinopathy (CSC) and SML safety profile. We conducted a prospective study including 31 fovea-involving CSC patients. The natural course was observed for the first 3 months, SML was performed at 3 months, and SML effectiveness was observed at 6 months. At all three clinical visits, optical coherence tomography (OCT), best corrected visual acuity (BCVA), contrast sensitivity (CS) in five spatial frequencies (1.5, 3.0, 6.0, 12.0, and 18.0 cycles per degree (cpd)), microperimetry (MP), and multifocal electroretinography (mfERG) were performed. The SML safety profile was evaluated with functional and morphological parameters. In the cohort of all CSC patients treated with SML, the statistically significant average improvement was observed in BCVA (*p* = 0.007), CS-1.5 (*p* = 0.020), CS-3.0 (*p* = 0.050), CS-12.0 (*p* < 0.001), CS-18.0 (*p* = 0.002), CS (CS-A) (*p* < 0.001), MP in the central ring (MP-C) (*p* = 0.020), peripheral ring (MP-P) (*p* = 0.042), and average retinal sensitivity (MP-A) (*p* = 0.010). After the SML treatment, mean changes in mfERG amplitudes and implicit times in our cohort were not statistically significant. No morphological or functional adverse effects of SML treatment were observed. SML treatment in persistent CSC episodes leads to significant functional improvement and has an excellent safety profile.

## 1. Introduction

Central serous chorioretinopathy (CSC) is a chorioretinal disease in the pachychoroid disease spectrum associated with retinal pigment epithelium (RPE) irregularities and subretinal fluid (SRF) accumulation [1]. It has a favorable prognosis with a spontaneous SRF resolution in 51.6% of patients within the first 3 months [1]. Acute CSC is characterized by SRF resolution within the first 3 months and best corrected visual acuity (BCVA) recovery to the pre-episode levels in the majority of patients [2]. More precise functional tests such as contrast sensitivity (CS) [3,4], microperimetry (MP) [5], and multifocal electroretinography (mfERG) [6,7] revealed impaired visual function even after SRF resolution. In persistent CSC, SRF persists for at least 3 months; however, there are no widespread RPE alterations or outer retinal layers atrophy [8]. A chronic CSC episode lasts more than 6 months and is associated with widespread RPE atrophy and severe vision loss leading to legal blindness [9]. Various CSC treatment modalities such as half-dose/fluence photodynamic therapy (PDT) [10], laser photocoagulation of the leakage spot(s) [11], subthreshold micropulse laser (SML) treatment [10], and oral spironolactone/eplerenone treatment [12] have been described.

In the SML treatment, the laser pulse is divided into active periods, which heat up the RPE cells, and inactive periods, which cool off the RPE cells, and prevent protein denaturation [13]. Temperature measurements in the porcine RPE cell model showed a different course of temperature rise with the SML treatment than with the continuous wave laser treatment [14]. Morphological improvement after SML treatment for CSC is well established [13,15,16,17]. The aim of the treatment is to accelerate SRF resolution and limit visual function impairment [17]. Currently, there are no studies evaluating visual function improvement in CSC patients treated with SML using the following battery of several functional tests at once: BCVA, CS, MP, and mfERG. We are aware of two studies comparing CS and BCVA in acute CSC patients treated with SML to those that were only observed [18,19]. Patients in the SML treatment group had better BCVA [19] and CS [18,19] outcomes. Moreover, a study comparing mfERG amplitudes and implicit times in acute CSC patients with or without SML treatment showed improved amplitudes in the first and second rings in the treated group at 3 and 6 months [20]. The SML treatment strategy in this study was treatment to the leakage site only [20].

In acute CSC patients, observation only is recommended by the majority of clinicians due to the self-limiting natural course [17]. However, other authors reported that SML treatment can shorten an acute CSC episode duration and improve BCVA [21]. Due to a favorable natural course in acute CSC patients, any intervention needs to have an excellent safety profile [22]. Eplerenone and spironolactone treatment can cause gynecomastia and hyperkalemia [23]; PDT adverse effects are choroidal hypoperfusion, choroidal neovascularization (CNV), and RPE atrophy [24]; and focal laser photocoagulation can cause RPE atrophy and CNV [17,25]. SML has a good safety profile as there have been no ocular adverse effects described when using appropriate laser pulse parameters [15]. SML safety profile in CSC patients has been evaluated by observing only morphological parameters such as RPE and outer retinal layer atrophy [21,25]. Chhablani et al. reported improvement in SRF reduction with no retinal tissue damage on optical coherence tomography (OCT) when using eight different micropulse laser parameters in CSC patients in a real-world setting [26]. Studies in retinal conditions showed decreased retinal sensitivity [27] and mfERG responses [28] in areas without morphological changes on multimodal imaging. Therefore, SML treatment could potentially iatrogenically decrease visual function parameters without affecting the morphology of the outer retinal layers and the RPE.

The aim of the study was to evaluate visual function outcomes in persistent CSC patients after the SML treatment, using several functional tests besides BCVA. Moreover, we wanted to evaluate the safety of SML treatment by observing precise visual function parameters and morphological changes on multimodal imaging.

## 2. Materials and Methods

The prospective study, which was approved by The National Medical Ethics Committee of the Republic of Slovenia (0120-141/2018/4) and adhered to the Declaration of Helsinki, was conducted from 2018 to 2021 at the University Eye Hospital in Ljubljana. All included patients provided signed informed consent to participate in the study. We included consecutive patients with acute CSC in the study. Inclusion criteria were acute CSC episodes with subfoveal neurosensory retinal detachment and durations of fewer than 3 months; however, only patients with persistent SRF at the fovea 3 months after the episode onset were treated with SML and included in the analysis. Patients were included in the study regardless if leakage was subfoveal or extrafoveal, and if SRF accumulation was solitary or multifocal. Exclusion criteria were allergy to fluorescein and ocular pathology that would have affected visual function tests.

Patients were examined at presentation, at 3 months, and at 6 months after the onset of visual symptoms. After ocular clinical examination, multimodal imaging was performed, including OCT, fundus autofluorescence (FAF), angiography with fluorescein (FA), and indocyanine green (ICGA). All multimodal imaging was performed with the Spectralis ophthalmic imaging platform (Heidelberg Engineering, Inc., Heidelberg, Germany). On the same day, visual function of the affected eye was determined using four functional tests: BCVA, CS, MP, and mfERG.

All aforementioned morphological and functional tests were performed at all 3 clinical visits except FA and ICGA, which were performed at presentation only. The natural course was observed in all patients for the first 3 months. At 3 months, patients with SRF fluid persistence 3 mm around the fovea were treated with the SML treatment. At 6 months, the SML treatment effectiveness was determined on the basis of morphological response to the treatment. Patients with persistent SRF at the fovea were grouped into the poor response group (pSML), while patients with complete SRF resolution at the fovea were grouped into the good response group (gSML).

Macular volume (MV) was determined as a sum of five subfields within 3 mm in the ETDRS grid, and central retinal thickness (CRT) was automatically determined by OCT software within 1 mm from the foveola.

BCVA was determined with the standard protocol using the Early Treatment Diabetic Retinopathy Study (ETDRS) visual acuity chart (Precision Vision, Woodstock, IL, USA). LogMAR values were used for the statistical analysis [29].

CS measurements were obtained in five different spatial frequencies (1.5, 3.0, 6.0, 12.0, and 18.0 cycles per degree (cpd)) using the FACT chart (Stereooptical CO, Chicago, IL, USA) and converted to logMAR values [30]. Average CS was calculated from five spatial frequencies.

Retinal sensitivity was measured with MP (Nidek Technologies, MP1, 2002, Padua, Italy) using a radial grid (45 test points, central 12° around the fovea) with a 4-2 staircase strategy, stimulus intensity 0–20 db, stimulus size Goldmann III, and projection time 0.2 s [31]. A red cross in the central 1° around the fovea was used as a fixation target [31,32]. Average retinal sensitivity (MP-A) was determined using all 45 test points, retinal sensitivity in the central ring (MP-C) using 13 central test points (4°), and retinal sensitivity in the paracentral ring (MP-P) using paracentral 32 test points (4°–12°).

The mfERG recordings followed the standard of the International Society for Clinical Electrophysiology of Vision (ISCEV) [33]. For measurement purposes, a Hawlina-Konec (HK) electrode was positioned in the lower conjunctival fornix [34], a reference Ag/AgCl electrode on the skin behind the temporal orbital rim, and a ground electrode was placed on the glabella. Stimulation cathode-ray tube screen (RETI port, Roland Consult, Germany) was used to display the stimulus, which consisted of alternating fields of black and white hexagons. The stimulus measured responses in an area of retina up to 30° from the fovea. Average amplitudes densities and implicit times from eight cycles of stimulation were determined from the ring around the fovea (1st ring) to the more peripheral rings (2nd–5th ring) [33].

SML treatment was conducted with a 577 nm laser (Supra Scan 577; Quantel Medical, Cournon d’Auvergne, France) using laser spots in a high-density and confluent fashion under the area of neurosensory retinal detachment. Foveal laser treatment was not avoided, and leakage site was not treated differently than other RPE under the neurosensory retinal detachment. Fixed parameters were used (250 mW, 5% duty cycle, 0.2 s, and 150 μm) without titration [35].

SML treatment safety was evaluated morphologically using OCT and FAF. Structural damage on OCT would be seen as atrophy of the outer retinal layers and the RPE, and on FAF as hypo autofluorescence in the area of previous treatment. Functional safety was evaluated by comparing the gSML group with normative values, and the gSML group with the group with spontaneous CSC resolution.

### Statistical Analysis

The Wilcoxon signed-rank test was used to assess the statistical significance of the difference (in the underlying distribution) between paired variables at presentation and second clinical visit (*p*_1_, at 3 months), and between paired variables at the second and the third clinical visit (*p*_2_, at 6 months). The Wilcoxon–Mann–Whitney test was used to assess the statistical significance of the difference (in the underlying distributions) between independent groups. The differences were deemed significant for two-sided *p*-values below 0.05. IBM SPSS Statistics for Windows, version 28 (IBM Corp., Armonk, N.Y., USA) was used for the implementation.

## 3. Results

Out of 50 patients with acute CSC, 31 patients had persistent SRF at 3 months and were treated with the SML treatment. Out of the 31 treated patients, 15 showed a good response (gSML) to treatment, while 16 patients responded poorly (pSML). Average baseline morphological characteristics were similar in the gSML and pSML groups. Table 1 presents visual function parameters, MV, and CRT in the cohort of all treated CSC patients at presentation, just before the SML treatment (at 3 months), and at 6 months.

In the cohort of treated patients, the average improvement in BCVA was statistically significant only after the SML treatment, i.e., from 3 to 6 months (*p*_2_ = 0.007). We observed statistically significant average improvements both from baseline to 3 months (p_1_) and from 3 to 6 months (*p*_2_) in CS-1.5 (*p*_1_ = 0.019; *p*_2_ = 0.020), CS-3.0 (*p*_1_ = 0.012; *p*_2_ = 0.050), and CS-A (*p*_1_ = 0.006; *p*_2_ < 0.001). In addition, we observed a statistically significant average improvement from baseline to 3 months in CS-6.0 (*p*_1_ = 0.005), and statistically significant average improvements from 3 to 6 months in CS-12.0 (*p*_2_ < 0.001) and CS-18.0 (*p*_2_ = 0.002).

We observed statistically significant average improvements both from baseline to 3 months (*p*_1_) and from 3 to 6 months (*p*_2_) in MP-C (*p*_1_ = 0.035; *p*_2_ = 0.001); MP-P (*p*_1_ = 0.044; *p*_2_ = 0.013); and MP-A (*p*_1_ = 0.033; *p*_2_ = 0.004). We observed statistically significant average improvements from baseline to 3 months in mfERG amplitudes in the first (*p*_1_ = 0.004) and second ring (*p*_1_ = 0.011); variables remained stable from 3 to 6 months, while the other average changes in mfERG variables were not statistically significant.

No structural RPE changes, which can be observed after continuous wave laser photocoagulation on OCT or FAF, were observed in any patient after the SML treatment.

MV, CRT, and visual function parameters in the gSML group are presented in Table 2. In this group, the average improvement in BCVA was statistically significant both from baseline to 3 months (*p*_1_ = 0.014) and from 3 to 6 months (*p*_2_ = 0.004). The average change in CS-1.5 was not significant, while there were significant average improvements after the SML treatment in CS-3.0 (*p*_2_ = 0.021), CS-6.0 (*p*_2_ = 0.006), CS-12.0 (*p*_2_ = 0.006), CS-18.0 (*p*_2_ = 0.004), and CS-A (*p*_2_ < 0.001). We observed statistically significant average improvements in MP-C both from baseline to 3 months (*p*_1_ = 0.022) and from 3 to 6 months (*p*_2_ = 0.001), while there were significant average improvements only after the SML treatment in MP-P (*p*_2_ = 0.013) and MP-A (*p*_2_ = 0.004). From baseline to 3 months, there were significant average changes in mfERG in the first ring (*p*_1_ = 0.042), while from 3 to 6 months, there were significant average changes in the amplitudes in the first (*p*_2_ = 0.010) and the second ring (*p*_2_ = 0.041), as well as the implicit time in the third ring (*p*_2_ = 0.016).

MV, CRT, and visual function parameters in the pSML group are presented in Table 3. We observed statistically significant average improvements from baseline to 3 months in CS-3.0 (*p*_1_ = 0.020), CS-6.0 (*p*_1_ = 0.044), mfERG amplitudes in the first (*p*_1_ = 0.045) and second ring (*p*_1_ = 0.031), and implicit time in the second ring (*p*_1_ = 0.017), while the only statistically significant average change from 3 to 6 months was that in CS-12.0 (*p*_2_ = 0.015). The other average changes were not statistically significant.

**Figure 1:**
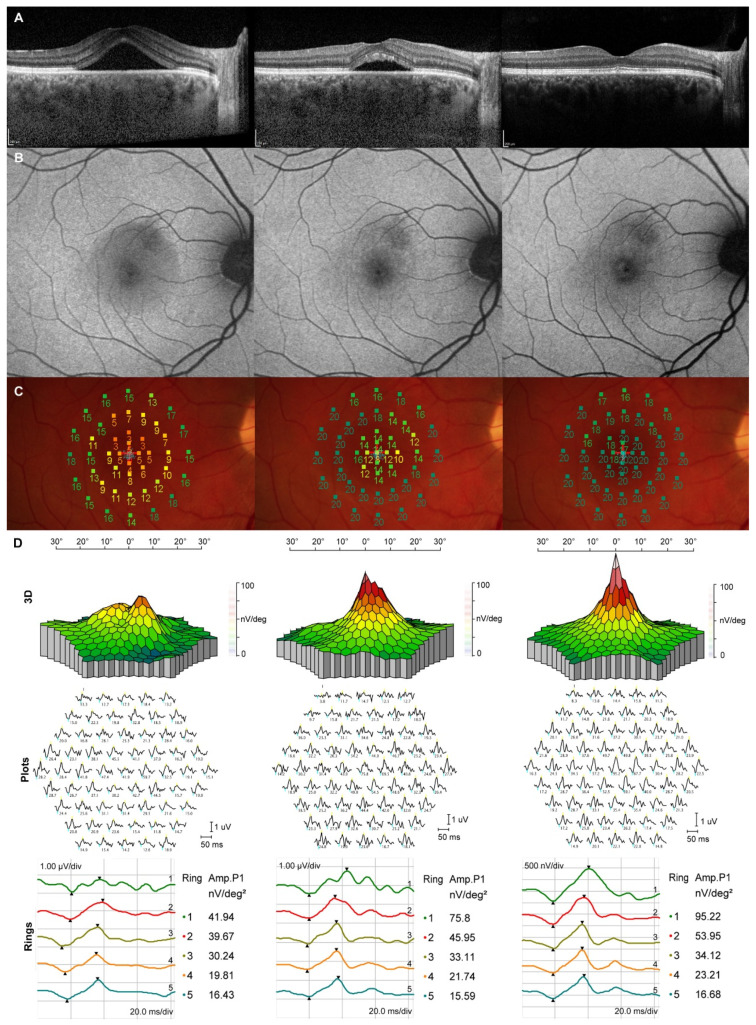
Morphological and functional longitudinal changes at baseline (left column), at 3 months when subthreshold micropulse laser (SML) treatment was performed (middle column), and at 6 months (right column). (**A**) Optical coherence tomography (OCT) shows partial spontaneous subretinal fluid (SRF) resolution from baseline to 3 months and complete SRF resolution after the SML treatment; (**B**) no visible SML scars on fundus autofluorescence; (**C**) microperimetry (MP) shows gradual retinal sensitivity improvement from baseline to 6 months; (**D**) Gradual improvement in multifocal electroretinogram (mfERG) amplitudes in the first and second ring.

Figure 1 depicts a patient with a persistent CSC episode and a good response to the SML treatment. At presentation, SRF accumulation was associated with significantly reduced retinal sensitivity, mfERG-A1, and mfERG-A2. At 3 months, partial spontaneous SRF reabsorption contributed to improved retinal sensitivity and mfERG amplitudes in the corresponding areas of SRF reabsorption. After the SML treatment at 6 months, SRF resolved completely, which resulted in retinal sensitivity and mfERG amplitudes improvement to normative values. No retinal laser spots were seen on the FAF.

## 4. Discussion

In our study, we observed significant visual function improvement after the SML treatment as measured with several precise functional tests. The statistically significant average improvement in visual function variables was observed in more variables in the gSML group, with complete SRF resolution, than in the pSML group, with persistent SRF. There were no signs of iatrogenically induced adverse effects associated with the SML treatment.

In our manuscript, we decided not to spare the fovea with SML treatment. A significant proportion of CSC patients have SRF accumulation and leakage limited only under the fovea. In these patients, treatment options are limited. Continuous laser photocoagulation at the fovea would lead to significant vision loss, and PDT could lead to chorioretinal atrophy and CNV formation with significant vision loss [24,25]. Therefore, trans-foveal SML could be the preferred treatment option in patients with limited SRF under the fovea due to the good safety profile. Moreover, maximizing the RPE surface area treated with SML could contribute to more extensive heat shock protein expression, which could ultimately lead to better RPE pumping ability.

In the cohort of all CSC-treated patients, the average decrease in MV was bigger from baseline to 3 months than from 3 to 6 months. Therefore, statistically significant average improvements in BCVA, CS, MP, and mfERG amplitudes in the first and second rings were observed from baseline to 3 months, while from 3 to 6 months, the only statistically significant average improvements were in BCVA, CS, and MP. We observed differences between the gSML and the pSML groups in visual function improvements after the SML treatment. Namely, in the gSML group, there were statistically significant average improvements in BCVA, CS, MP, and mfERG after the SML treatment, while in the pSML group, there were almost no statistically significant average changes. Previous studies of acute CSC showed that visual function is impaired in the topographical area of SRF accumulation [36,37]. In the pSML group, SRF was persistent, and therefore, visual function improvement was limited. However, in the gSML group, complete SRF resolution resulted in significant visual function parameters improvement. As the SRF accumulation was mostly confined to the topographical area of the first and second mfERG rings in the gSML group, statistically significant average improvements in amplitude densities were only observed in those rings, while no statistically significant average changes were observed in the more peripheral rings. Therefore, visual function improvement after the treatment is closely associated with the SML therapeutic effect of SRF reabsorption [17].

An important limitation in the majority of CSC trials is the difficulty in determining if the improvement is due to the natural course or the treatment used in the study. Since the morphological improvement in the gSML group was noted at 3 months already, we are unable to determine if complete SRF resolution would occur without the SML treatment or if the SML treatment caused SRF resolution. Nevertheless, several previous studies have established SML effectiveness in CSC patients [13,15,16,21].

Studies showed that even in dry age-related retinal degeneration and inherited retinal dystrophies with no SRF accumulation, SML treatment improved retinal sensitivity and pattern electroretinography parameters [38,39]. The SML treatment paradigm is in retinal cell stimulation without causing any structural damage [40]. It downgrades retinal inflammation and helps with the blood–retinal barrier restoration [41]. Therefore, SML could lead to potentially improved retinal cell function, which could result in improved visual function parameters. The combination of SRF resolution and improved retinal function after the SML treatment could lead to improved visual function parameters.

On the other hand, we could speculate that SML treatment could cause functional damage without affecting the retinal structure, as seen in multimodal imaging. As studies looking at SML adverse effects observed only morphological changes [21,25], the decrease in functional parameters without morphological changes could be overlooked. We compared the gSML group from this study and the sCSC group from our previous study (Table A1) [36]. The gSML and the sCSC groups had an acute CSC episode at baseline and episode resolution without SRF at 6 months. Patients in the gSML group had persistent SRF at 3 months and were treated with SML, while patients in the sCSC group had spontaneous SRF resolution at 3 months. If SML treatment had caused functional damage without morphological changes, functional outcomes at 6 months would have been worse in the gSML group in comparison with the sCSC group, which was not the case. Moreover, in the gSML group, mfERG parameters and retinal sensitivity at 6 months were close to the normative values [32,36], and we might speculate that further improvement in functional parameters would be observed with a longer follow-up. Therefore, trans-foveal SML treatment did not cause visual function impairment, and no structural changes were observed on OCT and FAF. This supports the case that the trans-foveal SML treatment with our fixed parameters is safe.

An important limitation when comparing results from studies evaluating morphological and functional outcomes in CSC patients treated with SML is the different treatment protocols. In some studies, fixed laser spot parameters were used [42], while other studies used various titration methods [21]. Moreover, different duty cycles were used, usually ranging from 5 to 15% [13]. In some studies, only the area with focal leakage, as seen on angiography, was treated [20], while in others, all areas under the neurosensory detachment were treated [18]. We believe that the SML treatment effect is in improving the RPE pump capacity; therefore, we treated our patients with high-density laser spots under the area of neurosensory detachment without sparing the fovea. Maltsev et al. reported good results in treating CSC patients with focal laser photocoagulation without FA [43]. With the treatment approach used in our study, which consisted of SML treatment under the whole area of neurosensory retinal detachment, FA would not be strictly necessary either. As described in the methods, we opted for fixed laser spot parameters instead of using a titration method. We used laser spot parameters (250 mW, 5% duty cycle, 0.2 s, 150 μm) that are already used widely as reported in the literature and showed similar effectiveness as SML treatment with a titration method [44]. However, fixed laser spot parameters might result in undertreatment, especially in patients with more extensive SRF accumulation [22]. In continuous wave laser photocoagulation, the same laser power was associated with worse laser uptake in an edematous retina than in a non-edematous retina [45]. A study showed that SML effectiveness (fixed parameters) was worse in CSC patients with higher maximal SRF [22]. Therefore, although fixed parameters might contribute to a better safety profile, this could also lead to undertreatment due to the inadequate thermal activation of the RPE.

In chronic CSC, in which treatment is necessary, superior anatomical outcomes were observed with the half-dose PDT in comparison with the high-density SML in the PLACE trial [10] and eplerenone in the SPECTRA trial [46]; however, functional outcomes (BCVA) were similar at the final evaluation in both trials. In acute CSC, half-dose PDT and SML treatment resulted in similar morphological and functional outcomes [47]. Half-dose/fluence PDT treatment is associated with several side effects [17], high cost, and limited availability [48]. Moreover, recently, there has been a worldwide shortage of verteporfin [49], making more widely available treatment options with a good safety profile, such as SML, more attractive. Although the SML mechanism of action remains controversial, a study showed that SML induced increased heat shock protein expression in cultured ARPE-19 cells without RPE cell damage [50]. Other studies suggested that SML treatment suppresses the vascular endothelial growth factor [35,51] and promotes the up-regulation of the angiogenic inhibitor (PEDF) [51]. In acute CSC, SML was similarly effective as half-dose PDT and had a better safety profile [47]. As acute CSC has a favorable natural course, any treatment used needs to have an excellent safety profile [17]. When SML was compared to observation, faster acute CSC episode resolution and better visual outcomes were observed [19]. Therefore, SML could be the preferred treatment modality in acute CSC patients.

This is the first study that showed improved functional parameters, including mfERG amplitudes and retinal sensitivity, in CSC patients treated with trans-foveal SML with laser spots applied under the area of neurosensory detachment. Moreover, this is the first study evaluating trans-foveal SML safety in CSC patients not only with morphological parameters but also with several functional parameters at once.

The limitations of our study are the small number of patients, short follow-up time, and lack of a control group. Even though we could not prove an excellent safety profile with absolute certainty, no morphological iatrogenic changes, good functional results, and comparable functional results between the gSML and sCSC groups suggest an excellent safety profile.

## 5. Conclusions

In conclusion, trans-foveal SML treatment in persistent CSC episodes results in morphological and functional improvement. Taking an excellent safety profile into account, SML could be a treatment of choice for acute and persistent CSC patients.

## Figures and Tables

**Table 1:** Morphological and functional changes in the cohort of all patients (n = 31) treated with subthreshold micropulse laser (SML).

	Baseline	3 Months (SML)	6 Months	*p*_1_	*p*_2_
MV, mm^3^, $\bar{x}$ ± SD	2.86 ± 0.66	2.55 ± 0.25	2.49 ± 0.33	**0.018**	0.247
CRT, μm, $\bar{x}$ ± SD	423 ± 135	358 ± 82	319 ± 94	**0.008**	**0.044**
BCVA, logmar, $\bar{x}$ ± SD	0.19 ± 0.16	0.16 ± 0.15	0.09 ± 0.14	0.090	**0.007**
CS-1.5, logmar, $\bar{x}$ ± SD	1.46 ± 0.13	1.51 ± 0.14	1.59 ± 0.18	**0.019**	**0.020**
CS-3.0, logmar, $\bar{x}$ ± SD	1.51 ± 0.21	1.61 ± 0.19	1.69 ± 0.21	**0.012**	**0.050**
CS-6.0, logmar, $\bar{x}$ ± SD	1.18 ± 0.56	1.43 ± 0.43	1.47 ± 0.61	**0.005**	0.121
CS-12.0, logmar, $\bar{x}$ ± SD	0.5 ± 0.61	0.67 ± 0.63	1.02 ± 0.58	0.180	**<0.001**
CS-18.0, logmar, $\bar{x}$ ± SD	0.16 ± 0.35	0.3 ± 0.42	0.62 ± 0.51	0.084	**0.002**
CS-A, logmar, $\bar{x}$ ± SD	0.97 ± 0.28	1.1 ± 0.29	1.28 ± 0.36	**0.006**	**<0.001**
MP-C, db, $\bar{x}$ ± SD	9.9 ± 5	11.5 ± 4.8	14.4 ± 5.3	**0.035**	**0.002**
MP-P, db, $\bar{x}$ ± SD	13.7 ± 4.2	14.8 ± 4	16.3 ± 3.2	**0.044**	**0.042**
MP-A, db, $\bar{x}$ ± SD	12.6 ± 4.3	13.9 ± 4.2	15.8 ± 3.8	**0.033**	**0.010**
mfERG-A1, nV/deg^2^, $\bar{x}$ ± SD	58.2 ± 22.5	68.1 ± 24	77.4 ± 28.1	**0.004**	0.065
mfERG-A2, nV/deg^2^, $\bar{x}$ ± SD	37.7 ± 10.4	40.5 ± 9.4	43.4 ± 9.9	**0.011**	0.244
mfERG-A3, nV/deg^2^, $\bar{x}$ ± SD	25.1 ± 5.4	25.9 ± 6.3	25.7 ± 5.3	0.247	0.769
mfERG-A4, nV/deg^2^, $\bar{x}$ ± SD	17 ± 3.5	16.9 ± 3.7	17.6 ± 3.6	0.794	0.272
mfERG-A5, nV/deg^2^, $\bar{x}$ ± SD	13 ± 2.8	12.7 ± 2.8	13.4 ± 3.1	0.350	0.267
mfERG-IT1, ms, $\bar{x}$ ± SD	41.6 ± 4.5	42.6 ± 4.7	43.3 ± 3	0.111	0.764
mfERG-IT2, ms, $\bar{x}$ ± SD	39.2 ± 1.7	38.7 ± 1.7	39.3 ± 1.3	0.243	0.206
mfERG-IT3, ms, $\bar{x}$ ± SD	37.5 ± 1.1	37.4 ± 1.1	37.2 ± 1.1	0.410	0.157
mfERG-IT4, ms, $\bar{x}$ ± SD	37.1 ± 1.1	37 ± 1	37 ± 1	0.320	0.878
mfERG-IT5, ms, $\bar{x}$ ± SD	37.6 ± 1	37.6 ± 1.1	37.6 ± 1.3	0.547	0.903

Macular volume (MV); central retinal thickness (CRT); best corrected visual acuity (BCVA); contrast sensitivity in spatial frequency 1.5 cycles per degree (cpd) (CS-1.5), 3.0 cpd (CS-3.0), 6.0 cpd (CS-6.0), 12.0 cpd (CS-12.0), 18.0 cpd (CS-18.0), and average cpd (CS-A); retinal sensitivity in central ring (MP-C); retinal sensitivity in paracentral ring (MP-P); average retinal sensitivity (MP-A); average amplitude density on multifocal electroretinogram in 1st ring (mfERG-A1), 2nd ring (mfERG-A2), 3rd ring (mfERG-A3), 4th ring (mfERG-A4), and 5th ring (mfERG-A5); average implicit time on multifocal electroretinogram in 1st ring (mfERG-IT1), 2nd ring (mfERG-IT2), 3rd ring (mfERG-IT3), 4th ring (mfERG-IT4), and 5th ring (mfERG-IT5); statistical significance of the average paired differences between functional parameters at presentation and just before the SML (at 3 months) (*p*_1_); and statistical significance of the average paired differences between functional parameters just before the SML (at 3 months) and at 6 months (*p*_2_).

**Table 2:** Morphological and functional changes in the subcohort of patients (n = 15) with good response to subthreshold micropulse laser (gSML).

	Baseline	3 Months (SML)	6 Months	*p*_1_	*p*_2_
MV, mm^3^, $\bar{x}$ ± SD	2.84 ± 0.59	2.52 ± 0.15	2.3 ± 0.13	**0.014**	**0.001**
CRT, μm, $\bar{x}$ ± SD	409 ± 130	333 ± 52	256 ± 21	**0.014**	**<0.001**
BCVA, logmar, $\bar{x}$ ± SD	0.18 ± 0.16	0.12 ± 0.15	0.02 ± 0.04	**0.014**	**0.004**
CS-1.5, logmar, $\bar{x}$ ± SD	1.51 ± 0.14	1.54 ± 0.13	1.64 ± 0.18	0.174	0.074
CS-3.0, logmar, $\bar{x}$ ± SD	1.56 ± 0.21	1.68 ± 0.16	1.8 ± 0.13	0.115	**0.021**
CS-6.0, logmar, $\bar{x}$ ± SD	1.28 ± 0.56	1.51 ± 0.46	1.74 ± 0.22	0.074	**0.006**
CS-12.0, logmar, $\bar{x}$ ± SD	0.57 ± 0.65	0.87 ± 0.65	1.22 ± 0.51	0.149	**0.006**
CS-18.0, logmar, $\bar{x}$ ± SD	0.18 ± 0.4	0.33 ± 0.44	0.81 ± 0.53	0.201	**0.004**
CS-A, logmar, $\bar{x}$ ± SD	1.02 ± 0.29	1.19 ± 0.28	1.44 ± 0.25	0.108	
MP-C, db, $\bar{x}$ ± SD	10.5 ± 5.3	13.1 ± 5.2	17.6 ± 2.7	**0.022**	**0.001**
MP-P, db, $\bar{x}$ ± SD	14.2 ± 4.1	16 ± 3.7	18.3 ± 1.7	0.080	**0.013**
MP-A, db, $\bar{x}$ ± SD	13.2 ± 4.3	15.2 ± 4	18.1 ± 1.9	0.080	**0.004**
mfERG-A1, nV/deg^2^, $\bar{x}$ ± SD	59.2 ± 20.4	69.6 ± 25.5	88.7 ± 27.1	**0.042**	**0.010**
mfERG-A2, nV/deg^2^, $\bar{x}$ ± SD	39.6 ± 11	41 ± 10.2	46.5 ± 10.7	0.234	**0.041**
mfERG-A3, nV/deg^2^, $\bar{x}$ ± SD	25.5 ± 6.5	26.3 ± 7.5	26.4 ± 6.9	0.293	0.955
mfERG-A4, nV/deg^2^, $\bar{x}$ ± SD	17.7 ± 4.1	17.5 ± 4.2	18.2 ± 4.3	0.529	0.691
mfERG-A5, nV/deg^2^, $\bar{x}$ ± SD	13.7 ± 3.3	13.2 ± 3.2	14.1 ± 3.6	0.262	0.330
mfERG-IT1, ms, $\bar{x}$ ± SD	41.8 ± 3.9	42.9 ± 4.2	42.1 ± 2.7	0.205	0.379
mfERG-IT2, ms, $\bar{x}$ ± SD	38.7 ± 1.1	39.2 ± 1.7	39.2 ± 1.1	0.270	0.915
mfERG-IT3, ms, $\bar{x}$ ± SD	37.7 ± 1.1	37.7 ± 0.9	37.1 ± 1	0.572	**0.016**
mfERG-IT4, ms, $\bar{x}$ ± SD	37.3 ± 1	37.3 ± 0.8	37 ± 1.1	1.000	0.439
mfERG-IT5, ms, $\bar{x}$ ± SD	37.6 ± 1	37.8 ± 0.8	37.4 ± 1.3	0.236	0.140

Macular volume (MV); central retinal thickness (CRT); best corrected visual acuity (BCVA); contrast sensitivity in spatial frequency 1.5 cycles per degree (cpd) (CS-1.5), 3.0 cpd (CS-3.0), 6.0 cpd (CS-6.0), 12.0 cpd (CS-12.0), 18.0 cpd (CS-18.0), and average cpd (CS-A); retinal sensitivity in central ring (MP-C); retinal sensitivity in paracentral ring (MP-P); average retinal sensitivity (MP-A); average amplitude density on multifocal electroretinogram in 1st ring (mfERG-A1), 2nd ring (mfERG-A2), 3rd ring (mfERG-A3), 4th ring (mfERG-A4), and 5th ring (mfERG-A5); average implicit time on multifocal electroretinogram in 1st ring (mfERG-IT1), 2nd ring (mfERG-IT2), 3rd ring (mfERG-IT3), 4th ring (mfERG-IT4), and 5th ring (mfERG-IT5); statistical significance of the average paired differences between functional parameters at presentation and just before the SML (at 3 months) (*p*_1_); and statistical significance of the average paired differences between functional parameters just before the SML (at 3 months) and at 6 months (*p*_2_).

**Table 3:** Morphological and functional changes in the subcohort of patients (n = 16) with poor response to subthreshold micropulse laser (pSML).

	Baseline	3 Months (SML)	6 Months	*p*_1_	*p*_2_
MV, mm^3^, $\bar{x}$ ± SD	2.87 ± 0.74	2.58 ± 0.32	2.68 ± 0.35	0.410	0.348
CRT, μm, $\bar{x}$ ± SD	436 ± 142	382 ± 99	378 ± 98	0.224	0.940
BCVA, logmar, $\bar{x}$ ± SD	0.2 ± 0.16	0.19 ± 0.15	0.17 ± 0.17	1.000	0.397
CS-1.5, logmar, $\bar{x}$ ± SD	1.42 ± 0.11	1.47 ± 0.13	1.55 ± 0.17	0.071	0.165
CS-3.0, logmar, $\bar{x}$ ± SD	1.47 ± 0.21	1.54 ± 0.2	1.58 ± 0.22	**0.020**	0.550
CS-6.0, logmar, $\bar{x}$ ± SD	1.09 ± 0.57	1.36 ± 0.41	1.21 ± 0.74	**0.044**	0.726
CS-12.0, logmar, $\bar{x}$ ± SD	0.43 ± 0.58	0.48 ± 0.57	0.83 ± 0.6	0.855	**0.015**
CS-18.0, logmar, $\bar{x}$ ± SD	0.15 ± 0.31	0.27 ± 0.42	0.45 ± 0.44	0.343	0.201
CS-A, logmar, $\bar{x}$ ± SD	0.91 ± 0.28	1.02 ± 0.27	1.12 ± 0.39	0.056	0.148
MP-C, db, $\bar{x}$ ± SD	9.3 ± 4.8	10 ± 4.1	11.2 ± 5.4	0.289	0.489
MP-P, db, $\bar{x}$ ± SD	13.2 ± 4.3	13.7 ± 4.1	14.4 ± 3.3	0.230	0.826
MP-A, db, $\bar{x}$ ± SD	12 ± 4.3	12.6 ± 4	13.5 ± 3.8	0.255	0.733
mfERG-A1, nV/deg^2^, $\bar{x}$ ± SD	57.2 ± 25	66.7 ± 23.1	66.8 ± 25.4	**0.045**	0.980
mfERG-A2, nV/deg^2^, $\bar{x}$ ± SD	35.9 ± 9.8	40.1 ± 9	40.5 ± 8.4	**0.031**	0.782
mfERG-A3, nV/deg^2^, $\bar{x}$ ± SD	24.7 ± 4.3	25.5 ± 5.2	25 ± 3.2	0.610	0.782
mfERG-A4, nV/deg^2^, $\bar{x}$ ± SD	16.3 ± 2.7	16.4 ± 3.2	17 ± 2.9	0.784	0.205
mfERG-A5, nV/deg^2^, $\bar{x}$ ± SD	12.4 ± 2.1	12.3 ± 2.3	12.7 ± 2.5	0.814	0.642
mfERG-IT1, ms, $\bar{x}$ ± SD	41.5 ± 5.1	42.3 ± 5.2	44.4 ± 2.9	0.284	0.208
mfERG-IT2, ms, $\bar{x}$ ± SD	39.7 ± 2	38.2 ± 1.7	39.4 ± 1.5	**0.017**	0.081
mfERG-IT3, ms, $\bar{x}$ ± SD	37.4 ± 1	37.1 ± 1.1	37.2 ± 1.1	0.071	0.592
mfERG-IT4, ms, $\bar{x}$ ± SD	37 ± 1.1	36.8 ± 1.2	37 ± 1.1	0.098	0.342
mfERG-IT5, ms, $\bar{x}$ ± SD	37.6 ± 1.1	37.5 ± 1.2	37.7 ± 1.4	0.595	0.125

Macular volume (MV); central retinal thickness (CRT); best corrected visual acuity (BCVA); contrast sensitivity in spatial frequency 1.5 cycles per degree (cpd) (CS-1.5), 3.0 cpd (CS-3.0), 6.0 cpd (CS-6.0), 12.0 cpd (CS-12.0), 18.0 cpd (CS-18.0), and average cpd (CS-A); retinal sensitivity in central ring (MP-C); retinal sensitivity in paracentral ring (MP-P); average retinal sensitivity (MP-A); average amplitude density on multifocal electroretinogram in 1st ring (mfERG-A1), 2nd ring (mfERG-A2), 3rd ring (mfERG-A3), 4th ring (mfERG-A4), and 5th ring (mfERG-A5); average implicit time on multifocal electroretinogram in 1st ring (mfERG-IT1), 2nd ring (mfERG-IT2), 3rd ring (mfERG-IT3), 4th ring (mfERG-IT4), and 5th ring (mfERG-IT5); statistical significance of the average paired differences between functional parameters at presentation and just before the SML (at 3 months) (*p*_1_); and statistical significance of the average paired differences between functional parameters just before the SML (at 3 months) and at 6 months (*p*_2_).

## Data Availability

The data presented in this study are available on request from the corresponding author. The data are not publicly available due to privacy restrictions.

## Appendix A

**Table A1:** Functional outcomes at 6 months in the good response CSC group (gSML groups) and spontaneous resolution CSC group (sCSC group) [36].

	gSML	sCSC	*p*
BCVA, logmar, $\bar{x}$ ± SD	0.02 ± 0.04	0.03 ± 0.06	0.520
CS-1.5, logmar, $\bar{x}$ ± SD	1.64 ± 0.18	1.71 ± 0.19	0.210
CS-3.0, logmar, $\bar{x}$ ± SD	1.8 ± 0.13	1.84 ± 0.22	0.802
CS-6.0, logmar, $\bar{x}$ ± SD	1.74 ± 0.22	1.77 ± 0.27	0.358
CS-12.0, logmar, $\bar{x}$ ± SD	1.22 ± 0.51	1.25 ± 0.62	0.433
CS-18.0, logmar, $\bar{x}$ ± SD	0.81 ± 0.53	0.83 ± 0.59	0.923
CS-A, logmar, $\bar{x}$ ± SD	1.44 ± 0.25	1.48 ± 0.34	0.520
MP-A, db, $\bar{x}$ ± SD	18.1 ± 1.88	17.74 ± 2.38	0.880
MP-C, db, $\bar{x}$ ± SD	17.6 ± 2.72	17.22 ± 3.16	1.000
MP-P, db, $\bar{x}$ ± SD	18.29 ± 1.66	17.96 ± 2.11	1.000
mfERG-A1, nV/deg^2^, $\bar{x}$ ± SD	88.66 ± 27.13	99.69 ± 27.63	0.317
mfERG-A2, nV/deg^2^, $\bar{x}$ ± SD	46.5 ± 10.72	49.2 ± 10.38	0.623
mfERG-A3, nV/deg^2^, $\bar{x}$ ± SD	26.39 ± 6.86	27.91 ± 5.45	0.558
mfERG-A4, nV/deg^2^, $\bar{x}$ ± SD	18.25 ± 4.29	18.62 ± 3.29	0.985
mfERG-A5, nV/deg^2^, $\bar{x}$ ± SD	14.12 ± 3.61	14.16 ± 2.95	0.940
mfERG-IT1, ms, $\bar{x}$ ± SD	42.09 ± 2.73	42.19 ± 2.38	0.834
mfERG-IT2, ms, $\bar{x}$ ± SD	39.25 ± 1.14	38.3 ± 1.33	**0.031**
mfERG-IT3, ms, $\bar{x}$ ± SD	37.11 ± 1.03	36.67 ± 1.36	0.240
mfERG-IT4, ms, $\bar{x}$ ± SD	36.99 ± 1.05	36.25 ± 1.26	0.210
mfERG-IT5, ms, $\bar{x}$ ± SD	37.37 ± 1.27	36.88 ± 1.45	0.559

Best corrected visual acuity (BCVA); contrast sensitivity in spatial frequency 1.5 cycles per degree (cpd) (CS-1.5), 3.0 cpd (CS-3.0), 6.0 cpd (CS-6.0), 12.0 cpd (CS-12.0), 18.0 cpd (CS-18.0), and average cpd (CS-A); retinal sensitivity in central ring (MP-C); retinal sensitivity in paracentral ring (MP-P); average retinal sensitivity (MP-A); average amplitude density on multifocal electroretinogram in 1st ring (mfERG-A1), 2nd ring (mfERG-A2), 3rd ring (mfERG-A3), 4th ring (mfERG-A4), and 5th ring (mfERG-A5); average implicit time on multifocal electroretinogram in 1st ring (mfERG-IT1), 2nd ring (mfERG-IT2), 3rd ring (mfERG-IT3), 4th ring (mfERG-IT4), and 5th ring (mfERG-IT5); and statistical significance between the gSML and sCSC groups (p). The single occurring *p*-value below 0.05 is considered statistically significant and is in bold.
